# Evidence for Oncolytic Virotherapy: Where Have We Got to and Where Are We Going?

**DOI:** 10.3390/v7122938

**Published:** 2015-12-02

**Authors:** Samantha Turnbull, Emma J. West, Karen J. Scott, Elizabeth Appleton, Alan Melcher, Christy Ralph

**Affiliations:** Leeds Institute of Cancer and Pathology, University of Leeds, Leeds LS2 9JT, UK; s.j.turnbull@leeds.ac.uk (S.T.); e.j.west@leeds.ac.uk (E.J.W.); k.j.scott@leeds.ac.uk (K.J.S.); elizabethappleton@doctors.org.uk (E.A.); a.a.melcher@leeds.ac.uk (A.M.)

**Keywords:** oncolytic virus, immunotherapy, reovirus, vaccinia, HSV, clinical trials

## Abstract

The last few years have seen an increased interest in immunotherapy in the treatment of malignant disease. In particular, there has been significant enthusiasm for oncolytic virotherapy, with a large amount of pre-clinical data showing promise in animal models in a wide range of tumour types. How do we move forward into the clinical setting and translate something which has such potential into meaningful clinical outcomes? Here, we review how the field of oncolytic virotherapy has developed thus far and what the future may hold.

## 1. Introduction

Even a brief search of the literature reveals a plethora of articles about the use of oncolytic viruses (OVs) in the treatment of cancer. For every virus, in almost every tumour type, there are robust data from laboratories around the world showing the potential of these agents in initiating tumour cell death. First reports of oncolytic viral therapy date back to the 1950s—using the intriguingly-named Bunyamwera, West Nile, and Semliki Forest viruses [1]. Despite this, the first phase III trial of a viral-based therapy for melanoma has only just been reported [2]. The field has now expanded to include DNA viruses (herpes simplex virus (HSV), adenovirus, vaccinia virus (VV)), RNA viruses (coxsackie virus, respiratory and enteric orphan virus (reovirus), measles virus (MV)), and genetically modified viruses such as talimogene laherparepvec (HSV expressing granulocyte-macrophage colony stimulating factor (GM-CSF), known as T-VEC). While many trials have focused on clinical endpoints, such as safety, response rate (RR), and overall survival (OS), very few have examined immune translational readouts (Table 1 and Table 2).

**Table 1:** A selection of recently published clinical trials of oncolytic viruses.

Virus	Delivery	Phase	Disease	*n*	Description	Year	Reference
Adenovirus	*i.t.*	Ib/II	Advanced solid tumours	60 (first part) 115 (second part)	Evaluation of immunological response to and safety/survival of patients treated with single *vs.* serial doses of various oncolytic viruses	2013	[3]
	Subcutaneous *(s.c.)*	I/II	CRC	32	Ad5 (coding for CEA) dose escalation study and expansion cohort for safety, tolerability and immune responses	2013	[4]
	*i.t.*	I	Advanced solid tumours	9	CD40L-expressing replicating adenovirus in patients with solid tumours (immune reponse and safety evaluation)	2012	[5]
	Intravesicular	I	Bladder cancer	35	Dose finding study of GM-CSF-expressing adenovirus (CG0070)	2012	[6]
Coxsackie	*i.t.*	II	Melanoma	57	Evaluation of local and distant tumour responses in patients with metastatic disease	2014	[7]
Reovirus	*i.v.*	Ib	Colorectal cancer	10	Proof of principle study in patients undergoing resection of liver metastases	2012	[8]
	*i.v.*	I	Advanced solid tumours	36	Evaluation of cyclophosphamide in optimising OV delivery	2015	[9]
	*i.t.*	I	Glioma	15	Dose finding and safety study of 72 h *i.t.* infusion	2014	[10]
	*i.v.*	Ib	Recurrent glioma/metastatic brain tumours	12	Evaluation of delivery and immunological response to OV prior to surgery	2014	[11]
	*i.v.*	I/II	Advanced solid tumours and head and neck cancer	31	Dose finding study in combination with carboplatin and paclitaxel	2012	[12]
	*i.v.*	I	Advanced solid tumours	16	Combination of OV and gemcitabine	2011	[13]
	*i.v.*	I	Advanced solid tumours	24	Combination of OV and docetaxel	2010	[14]
	*i.t.*	I	Advanced solid tumours	23	Combination of OV and radiotherapy	2010	[15]
	*i.v.*	II	Melanoma	21	Evaluation of tolerability and efficacy	2012	[16]
HSV	*i.t.*	III	Melanoma	436	HSV encoding GM-CSF (Talimogene Laherparepvec; T-VEC) *vs.* GM-CSF in patients with unresectable disease	2015	[2]
	*i.t.*	Ib	Melanoma	19 (ongoing)	Combination of T-VEC and the checkpoint inhibitor ipilimumab	2014	[17]
	*i.t.*	I	GBM	9	Combination of OV with radiation for recurrent GBM	2014	[18]
	*i.t.*	I/II	Head and neck cancer	17	Combination of HSV encoding GM-CSF with radiotherapy and cisplatin prior to surgery	2010	[19]
Vaccinia	*i.t.*	I	HCC	30	Dose-finding study of vaccinia encoding GM-CSF (pexastimogene devacirepvec; JX-594)	2013	[20]
Vaccinia/Fowlpox	*s.c.*	I	Prostate cancer	30	Combination of ipilimumab and a vaccine (PROSTVAC-VF) targeting PSA	2012	[21]
	*s.c.*	II	Prostate cancer	125	PROSTVAC-VF plus GM-CSF *vs.* placebo	2010	[22]
VSV	*i.t.*	II	HCC and cholangiocarcinoma	12 (ongoing)	VSV with beta interferon insert in patients with metastatic liver cancers	2015	[23]

**Table 2:** A selection of currently open clinical trials registered with the national cancer institute.

Virus	Delivery	Phase	Disease	Description	NCT Identifier
Coxsackie	*i.t.*	I	Melanoma	Combination of CAVATAK^®^ with ipilimumab or pembrolizumab	02307149
					02565992
HSV	*i.t.*	Ib	Melanoma	Combination of ipilimumab or pembrolizumab with or without T-VEC	01740297
					02263508
	Intrapleural	I/II	Mesothelioma	Evaluating the biological effects and safety profile	01721018
	*i.t.*	I	Brain tumours	Dose-finding and safety study of G207 with or without a single dose of radiotherapy in children with recurrent brain tumours	02457845
Measles	Intrapleural	I	Mesothelioma	Dose-finding and safety study	01503177
	*i.v.*	II	Myeloma	Combination of OV and cyclophosphamide	02192775
	*i.t.*	I	Head and neck cancer	Dose-finding and safety study	01846091
Parvovirus	*i.t./i.v.*	I/IIa	GBM	Combination of *i.t.* or *i.v.* OV followed by tumour cavity injection at surgery	01301430
Vaccinia/Fowlpox	*s.c.*	III	Prostate Cancer	PROSTVAC-VF + GM-CSF	01322490

## 2. Mechanism of Action: Direct Cytotoxicity or Immune Activation?

### 2.1. Direct Oncolysis of Malignant Cells

The importance of *in vitro* work in understanding the method of action of OV therapy is not in question. If we understand the mechanism of oncolysis it may help to guide us to the most appropriate clinical application. It has been postulated for some time that reovirus, for example, can act preferentially on cells which have activating *Ras* mutations [24,25,26]. The Ras (rat sarcoma) proteins are integral to intracellular signal transduction pathways which control cell growth, replication, angiogenesis, and apoptosis. These pathways can be activated by cell surface receptors (receptor tyrosine kinases, RTK), such as the epidermal growth factor receptor (EGFR, also known as human epidermal growth factor-1, HER-1). Binding of growth factors in the cell microenvironment to these receptors triggers an intracellular cascade. Mutations in this pathway lead to increased signalling in the absence of stimuli and the regulation of cell survival becomes abnormal [27,28].

The mechanism for preferential targeting and viral replication of cells with *Ras* activation seems to be via double-stranded RNA (dsRNA)-activated protein kinase (PKR) [25]. Reovirus infection of cells (and the presence of dsRNA) leads to activation of PKR which, in turn, phosphorylates and inactivates eukaryotic translation initiation factor 2α (eIF-2α) resulting in inhibition of viral transcription. In *Ras*-activated/mutated cells, PKR activation is blocked and viral replication is able to continue. There would, therefore, seem to be a strong rationale for the use of reovirus in tumours with a high incidence of *Ras* mutations [29]. However, more recently, this hypothesis has been called into question and the exact mechanisms of direct cytotoxicity may be more complex [30].

Similarly, Onyx-015, a human adenovirus genetically modified without the *E1B 55K* gene, is able to replicate preferentially in cells with deficiency of the *p53* tumour suppressor gene. Consequently, it has increased oncolytic activity against cells which have *p53* mutations in an *in vivo* mouse model [31]. However, this is not observed in the clinic setting: patients with *p53* mutations treated with Onyx-015 had a lower OS when compared to wild-type *p53*, although in such a small number of patients this was not significant [32].

Vesicular stomatitis virus (VSV) is one of a number of OVs which are thought to be more cytotoxic to cells which have impaired defence mechanisms against pathogens: in this case, the interferon (IFN) response [33,34]. OVs can also preferentially target cells with increased expression of specific cell-surface receptors compared to normal host cells, such as MV and CD46 [35,36]. In practice, as we have already seen, the mechanisms of direct oncolysis by OVs may be multiple, complex, and vary with virus, tumour, and the interplay between them [37,38].

### 2.2. Oncolytic Viruses as an Immunotherapy

By better understanding the mechanisms of OV therapy, we can not only gain insight into how to apply these agents clinically, but investigate who the likely responders may be. No matter how robust the model, we need to ensure it is applicable in the clinical setting to drive therapy forward. So far, there has been a dearth of knowledge about the immune response in such a setting but trials are now starting to reveal interesting data [8,39].

As well as a direct cytotoxic effect via apoptosis, necrosis or viral overload, there is increasing evidence that OVs stimulate an anti-tumour immune response [40,41,42]. This can be innate, driven by cytokine release causing migration of immune cells such as natural killer (NK) cells to the site of disease, or adaptive, via a response to tumour-associated antigens (TAA).

### 2.3. Innate Immune Response

*In vitro* treatment of monocyte-derived healthy donor dendritic cells (DCs) with reovirus can stimulate secretion of IFN-α, interleukin (IL)-6, IL-12p70, and tumour necrosis factor (TNF)-α. In turn, these activated DCs stimulate NK cells and T cells, leading to increased secretion of IFN-γ and more potent tumour cell cytotoxicity [42]. Myxoma virus [43] and parvovirus [44,45] have similarly been shown to induce NK cell-mediated death of glioma cell lines (myxoma), pancreatic and colorectal cancer (CRC) cell lines (parvovirus).

NK activation by reovirus has also been seen in chronic lymphocytic leukaemia (CLL) [46] and CRC patients [39]. Exposure of both healthy donor and patient NK cells to reovirus resulted in increased CD69 expression along with enhanced cytotoxicity towards relevant tumour cell targets, indicated by up-regulated CD107 expression (a marker of NK cell degranulation) [39,46]. Peripheral blood mononuclear cells (PBMCs) also secrete IFN-α, IFN-β and Regulated on Activation, Normal T cell Expressed and Secreted (RANTES) after treatment with reovirus. Blocking this type I IFN response reverses the effects of reovirus on both NK activation and cytotoxic degranulation [39,46]. Depletion of the CD14+ monocyte population within PBMCs similarly causes a decrease in IFN-α production and NK cell activity, suggesting a pivotal role for the monocyte population in this process [46].

### 2.4. Adaptive Immune Response

DCs co-cultured with reovirus-treated melanoma cell lines can prime cytotoxic T lymphocytes (CTLs) for an adaptive immune response against specific tumour targets [41]. Moreover, the ability of reovirus to generate an adaptive immune response against tumour antigen is separate from direct oncolysis. B16ova melanoma cells, which express low levels of the reovirus receptor junctional adhesion molecule-A (JAM-A), are resistant to direct reovirus oncolysis. Immunocompetent mice implanted with B16ova tumours were treated with reovirus, the splenocytes harvested and exposed to different cell lysates. IFN-γ production was increased with exposure to melanoma lysate antigens, indicating the activation of a specific anti-tumour response in the absence of direct cytotoxicity [47].

Treatment of tumour-bearing animals with serum from rabbits treated with pexastimogene devacirepvec (VV expressing GM-CSF, termed JX-594) led to improved survival, indicative of an adaptive antibody-mediated complement-dependent anti-cancer effect [48]. Cancer cell lines exposed to serum from patients treated with JX-594 showed decreased viability, particularly when these cells were similar to the patient’s original tumour. Serial treatments of patients with adenovirus expressing GM-CSF led to an increase in specific T cell responses against both virus and tumour [3]. These data support the hypothesis of an OV-induced specific antibody, as well as CTL, mediated anti-tumour response.

## 3. Evaluating the Immune Response to Oncolytic Therapy

There has always been concern about the application of phase I trial outcomes to the general patient population, given that these patients tend to be a selective group with a heterogenous disease burden who have usually been heavily pre-treated. The data in the clinical setting seems reassuring in this regard: the limited data suggests phase I patients can mount an immune response following treatment with OVs. Whether this can be extrapolated to patients treated with curative intent or the treatment naïve is unclear.

### 3.1. Neutralising Antibody Response

In a phase I trial of reovirus, an increasing neutralising antibody (NAb) response was seen during treatment, despite a high proportion of seropositivity at baseline [40], suggesting that patients are capable of mounting a humoral immune response to viral therapy. In patients with CRC receiving adenovirus (coding for carcinoembryonic antigen, CEA), over half the patients had NAbs at baseline yet, despite increasing levels, there was evidence of an antigen specific immune response to treatment [4], indicating that NAb levels may not be a barrier to successful therapy.

### 3.2. Immune Cell Populations

Peripheral blood immune cell subsets are affected by OVs: patients with advanced solid tumours treated with intravenous (*i.v.*) reovirus had lower CD4+ T cells at baseline, compared to relatively high populations of CD8+ T cells and NK cells [40]. Following treatment, there was an increase in all of these sub-populations but no obvious change in the numbers of regulatory T cells (Tregs). Interestingly, an increasing CD4+/CD8+ ratio has previously been postulated as a predicator of positive outcome in patients with metastatic melanoma receiving chemo-immunotherapy (combination cytotoxic chemotherapy plus IFN) [49].

Tumour infiltrating lymphocytes (TILs) have been shown to be prognostic in a number of different cancers ([50,51] and reviewed in [52,53,54]). Changes in tumour immune infiltrates before, during and after OV therapy are also of interest. Following intratumoural (*i.t.)* therapy with adenovirus expressing GM-CSF (ONCOS-102), there was increased infiltration of CD4+/CD8+ cells into the tumour microenvironment [55]. Moreover, *i.t.* therapy can initiate infiltration of immune cell populations into non-injected lesions, which may go some way to explain why *i.t.* OVs can affect both injected and non-injected tumours [56,57].

### 3.3. Cytokine Response: Do We Really Need Replicating Virus?

Following infection with reovirus, both human melanoma cell lines and primary melanoma cells produce increased amounts of IL-8 and RANTES, which can recruit immune cells into sites of inflammation [58,59]. In addition, IFN-β and IFN-γ-induced protein 10 (IP-10, also known as C-X-C motif chemokine 10, CXCL-10), another chemokine which triggers migration of effector immune cells, can be detected [59]. Associated with this, NK cells, DCs, and CTLs (primed against melanoma cell lines) all migrate towards tumour-conditioned media from reovirus-infected melanoma cells (reoTCM) in the absence of the virus itself. Down-regulation of the anti-inflammatory cytokine IL-10 (a Th2 cytokine) is also observed after therapy *in vitro* [58]. Moreover, a cytotoxic effect on previously reovirus-resistant cells treated with reoTCM is evident, in addition to DC and NK cell activation [58,59]. ReoTCM from melanoma cells is also able to support priming of CTLs by DCs against relevant tumour targets. This is associated with release of IFN-γ, IL-6 and TNF-α but not IL-10, supporting a possible T helper (Th) 1 immune response [41]. This suggests that reovirus infection produces an immune milieu that can elicit an immunogenic and cytotoxic response against tumour.

Intrahepatic injection of a genetically-modified adenovirus in patients with metastatic colon cancer induced a rise in the pro-inflammatory cytokines TNF-α, IFN-γ, and IL-6 [60]. In most of the patients treated, levels peaked before falling, followed by a modest rise in IL-10 levels. Interestingly, a patient who had a systemic inflammatory response to treatment had very high IL-6 and no increase in IL-10. Of note, these responses varied between and within patients with different cycles of treatment.

Similar effects have been seen clinically in an ovarian cancer patient treated with the adenovirus ONCOS-102 (expressing GM-CSF) [55] and adenovirus expressing CD40-ligand (CD40-L) [5]; increased IL-10 was again seen [3].

In mice treated with intraperitoneal (*i.p.*) HSV, as well as increased NK and CD8+ cells in the peritoneal fluid, there was also increased IP-10 and IFN-γ produced by DCs and monocytes in response to the virus. These chemokines may recruit immune cell populations to the tumour site; their production was abrogated by introducing anti-IFN-α or anti-IFN-β antibodies, suggesting a type 1 IFN-mediated mechanism [61].

In *in vitro* models, reovirus-induced cytotoxicity can be mediated by TNF-Related Apoptosis-Inducing Ligand (TRAIL), with increased release in response to infection and increased apoptosis of human embryonic kidney (HEK293) cells [62].

## 4. The “Neutralising Antibody” Problem

The NAb response to OVs was initially considered a barrier to be overcome, particularly when a high proportion of the population has been exposed to the virus in childhood (adenovirus and reovirus being particular examples). It has, however, become increasingly apparent that the interaction between virus, the host immune response, and NAbs is much more complex.

There was initially interest in combining OV with immunosuppressants, such as cyclophosphamide, with the aim of attenuating the anti-viral immune response, specifically production of NAbs, to enhance viral delivery to tumours. This showed some promise in the mouse; CD46 transgenic mice treated with MV and cyclophosphamide were shown to have significantly lower levels of anti-MV antibody [63]. In contrast, when *i.v.* cyclophosphamide was administered with reovirus to patients with advanced solid tumours, no significant inhibition of the NAb response was seen [9].

A number of hypotheses describe how OVs can survive despite a host immune response. Specifically, many studies have shown that virus may evade NAbs due to association with immune cells [8,9,64,65]. Reovirus-loaded human DCs can induce melanoma cell line death even in the presence of human serum containing NAbs via internalisation of the virus [64]. This could not be replicated with T cells, where the virus attaches to the cell surface. This phenomenon was observed in blood from patients undergoing resection of liver metastases following *i.v*. infusion of reovirus: there was evidence of viral carriage on PBMCs, platelets, and granulocytes but not in cell-free plasma [8]. Replication-competent virus was also found in tumour cells but not normal liver cells, suggesting that, by association with blood cell subsets, active reovirus can be specifically delivered to tumours despite the presence of NAbs (a process termed “hitch-hiking”) [39]. CD4+ and CD8+ T cells, NK cells, B cells and monocytes have all been found to be positive for the JAM-A receptor, which may be a potential mechanism of viral carriage *in vivo* [39]. Reovirus association with these cell populations was not, however, cytotoxic to them.

The complex role of NAbs was further investigated using adenovirus (encoding CEA) in patients with advanced CRC receiving increasing doses of virus. Over half the patients treated already had evidence of NAbs at baseline yet, despite this (and evidence of further increases in NAb levels over time), an antigen-specific cell-mediated immune response was observed [4].

In summary, these data indicate that, despite historical beliefs that NAbs would detrimentally impede delivery of virus to tumour, virus can still be detected in tumour tissue after *i.v.* therapy.

## 5. Delivery of Oncolytic Virotherapy

When it comes to how to deliver your OV of choice, the only option that is currently unavailable is also, ironically, the most convenient for the patient: oral therapy. Oral and *i.v.* delivery of chemotherapy has the obvious advantage of potentially reaching all sites of systemic disease. Alternative routes of delivery have been investigated to try and avoid NAbs, on the basis that they would neutralise the virus before it had a chance to have any effect.

Intratumoural injection of OV directly into malignant lesions may offer a solution. It has been reassuring to see that *i.t.* viral therapy can generate a systemic response, affecting even non-injected tumours [56,57]. T-VEC and JX-594 have both been given *i.t..* Both agents have shown disease response in injected and non-injected tumours and both have entered late-phase clinical trials [2,20,56,66].

However, *i.t.* administration is not possible in all patients. Intravenous delivery can, in theory, reach all disease sites but the virus must evade the host immune response to do so. Similarly, the oncolytic, cytotoxic, and immune response to a virus given *i.t.* will not be the same as when an identical virus is given *i.v*..

In murine models of non-small cell lung cancer and mesothelioma, HSV and adenovirus have been used intrapleurally with effects seen in terms of the bulk of disease and on malignant effusions [67,68,69]. MV was given *i.p.* in 21 patients with recurrent ovarian cancer in a phase I trial, resulting in falling CA-125 tumour markers (five patients) and stable disease (SD; 14 patients, median duration 92 days) [70]. Different strains of adenovirus have also been used intravesically in mouse [71] and human [6] models, where in a phase I trial the virus had an acceptable safety profile and there was evidence of viral replication and activity.

Targeted delivery of OV may also have benefit in localised disease. Vaccinia has been used in combination with TNF-α and melphalan, given via isolated limb perfusion. The blood supply to a limb is isolated with a tourniquet before treatment is administered *i.v..* In murine sarcoma, combination therapy, including vaccinia, led to delayed tumour growth and increased survival [72]. Convection-enhanced delivery (CED) has also been investigated *in vivo* in the brain. In patients with recurrent malignant glioma, reovirus was given over 72 h by slow infusion via intralesional catheters that had been placed surgically [10]. This approach circumvents the blood-brain barrier, which has been presumed to be an obstacle when treating intracranial disease. The results of a phase Ib translational study of reovirus given *i.v.* to patients prior to planned surgery for recurrent high grade primary or metastatic brain tumours is awaited [11]. It would, however, be hard to incorporate methods such as limb perfusion and CED into the clinic outside of intensive trial practice in a tertiary centre.

When investigating the best method of administration for OV delivery, consideration should be given to combining different routes. There is currently an ongoing phase I/IIa dose-finding and safety study investigating this in patients with recurrent glioblastoma multiforme (GBM). One group of patients will receive oncolytic parvovirus *i.t.* followed 10 days later, at surgery, by infiltration into the tumour cavity. The second group of patients will receive virus *i.v.* initially, again followed by tumour cavity injection at surgery (NCT01301430).

Early phase trials of *i.v.* reovirus have already shown evidence of replicating virus in surgically-resected samples [8,73]. Choosing an *i.t.* route of delivery has obvious downsides in the immediate exclusion of patients who do not have accessible disease. Thus, while alternative methods of delivery have been sought to evade a host immune response, the most obvious answer may turn out to be the simplest and most pragmatic (systemic *i.v.* administration), provided that therapeutic efficacy can be demonstrated*.*

## 6. Combination Therapy: Are Two Drugs Really Better than One?

The efficacy of single agent OVs has, until recently, been fairly modest. If we are considering using OVs as an adjunct to other agents, their action needs to be understood in order to develop rational treatment paradigms.

OVs have been combined with many other therapeutic agents in animal models. Any synergistic effect, either immunogenic or cytotoxic, will hopefully lead to increased therapeutic efficacy, including potentially increasing the sensitivity of tumour cells to chemo- or radio- therapy or targeting resistant cell populations. Drugs that increase activation of the tumour suppressor gene *p53* can, for example, increase the sensitivity of cells to reovirus-mediated oncolysis [74]. Viruses can also be used as a vector to deliver other agents to tumour cells. The challenge, as always, is to develop a practical, efficacious combination therapy, with limited combined toxicity.

### 6.1. Oncolytic Viruses and Chemotherapy

Adenovirus expressing CD40-L has been given in combination with 5-flurouracil (5-FU) chemotherapy, with evidence of efficacy when given intravesically in an immuno-competent mouse bladder tumour model. Interestingly, the same effect was not seen in nude immunosuppressed mice, suggesting that T cell-mediated immunity may have a role [71].

Reovirus has been combined with a number of chemotherapeutic agents in prostate cancer cell lines *in vitro*. *In vivo*, decreased tumour growth and increased survival in combination with docetaxel was observed [75].

In combination with cisplatin-paclitaxel, reovirus showed some pre-clinical efficacy in head and neck cancer cell lines, with increased tumour cell apoptosis and prolonged survival in a murine model [76]. This informed a phase I/II clinical trial of reovirus with carboplatin-paclitaxel chemotherapy in patients with advanced malignancy and, as an expansion cohort, patients with head and neck cancer [12]. There were no reports of dose-limiting toxicity (DLT), confirming that combination therapy can be well tolerated. The subsequent phase II trial in patients with head and neck cancer showed some evidence of efficacy: median OS was 7.1 months, *versus* the 3.5–4.5 months quoted in other studies of second line chemotherapy [12]. The results of a phase III trial to evaluate the initial findings are awaited.

Although the combination of OVs with cytotoxic chemotherapy has potential, the specific immune interactions need to be thoroughly investigated. There is now increased recognition that chemotherapy agents themselves may have immunomodulatory effects on B cell, macrophage, and NK cell responses and heterogeneous effects on the activity and maturation of DCs [77,78]. Gemcitabine has been shown to potentiate the oncolytic effect of reovirus through inhibition of myeloid-derived suppressor cells (MDSC), which can suppress an anti-tumour immune response [79]. Similarly, patients have been found to have a lower proportion of Tregs in PBMCs after receiving docetaxel chemotherapy for prostate cancer [80].

Toxicity of viral therapy has, for the most part, been self-limiting: flu-like illness, fever, diarrhoea, nausea, and vomiting. In a phase I dose escalation study of reovirus, levels of NAbs were suppressed when combined with gemcitabine [13]. Dosing was switched to once every three weeks, instead of the planned five consecutive days, after the first two patients experienced a DLT (hepatic transaminitis). The authors hypothesised the low levels of NAb could contribute to toxicity of reovirus. In contrast, when reovirus was given with docetaxel, there was no obvious effect on NAb levels [14]. Unlike in the gemcitabine study, the maximum tolerated dose (MTD) was not reached.

### 6.2. Oncolytic Virus and Checkpoint Inhibitors

Antibodies targeting cytotoxic T-lymphocyte antigen-4 (CTLA-4) and programmed death-1/programmed death ligand-1 (PD-1/PD-L1) are already being used clinically in a range of malignancies, including melanoma, where they have had very positive effects [81,82,83,84].

Melanoma antigen specific vaccines have been evaluated in early phase clinical trials in the adjuvant setting [85] and were found to increase PD-1 expression at the injection site. Similarly, there is emerging data that OVs can regulate the expression of PD1/PD-L1 on cancer cells [86], providing a strong rationale for combination therapy with checkpoint inhibitors.

In B16 murine melanoma models, MV encoding anti-CTLA-4 and anti-PD-1 enhanced therapy, delaying tumour progression and increasing survival [87]. Immune studies showed a lower percentage of Tregs and a higher proportion of CTLs within the treated tumour microenvironment. In the same model, the combination of anti-PD1 antibody and reovirus led to increased survival compared to either agent alone [88]. Here, anti-PD-1 treatment down-regulated Treg cell activity and increased effectiveness of NK cell-mediated lysis of malignant cells infected with reovirus.

A major concern in combining OV with immune checkpoint blockade is the potential to increase immune-mediated side effects, which can already be unpredictable and debilitating. In a phase I dose-escalation trial, the anti-CTLA-4 monoclonal antibody, ipilimumab, was combined with a poxvirus-based vaccine in patients with castrate-resistant prostate cancer [21]. The vaccine stimulated a T cell response specific to prostate specific antigen (PSA). The side effect profile did not seem to differ significantly over single agent therapy with ipilimumab. There remained, however, an almost 30% incidence of Common Toxicity Criteria for Adverse Events (CTCAE) grade-three toxicity. Interestingly, patients who were chemotherapy-naïve had a higher chance of a decrease in PSA. This again raises the possibility that patients who have previously been treated with chemotherapy might have an altered immune profile and may respond differently to immunotherapy.

Most recently, encouraging early data has been reported from combination T-VEC with anti-CTLA-4 antibody [17] and a phase III trial adding T-VEC to anti-PD-1 antibody is planned. 

### 6.3. Oncolytic Virus and Radiotherapy

*In vivo* and *in vitro* models of radiotherapy and reovirus (or other OVs) seem to support an enhanced response, with evidence of increased cytotoxicity when used in combination [89].

In a phase I trial of *i.t.* reovirus in patients with advanced cancer, participants also received five fractions of palliative radiotherapy to disease sites [15]; there were no DLTs. While mutations in the *Ras* gene are thought to confer relative resistance to radiotherapy, the cytotoxic effect of reovirus may be selective for cells with an activated *Ras* pathway [90]: the addition of reovirus should, therefore, have a synergistic effect on cell death when combined with radiotherapy.

HSV G207 has been given *i.t.* to patients with glioma prior to a single palliative fraction of radiotherapy [18]. Although this was a modest dose, there was evidence of radiological response (SD or partial response (PR)) in six of nine treated patients, also suggesting some synergistic activity. The presence of pre-existing antibodies to HSV was not predictive of response, once again indicating that the role of NAbs may be more complex than initially thought.

### 6.4. Oncolytic Virus and Chemo-radiotherapy

As OVs have shown promise in head and neck cancers [12], patients with stage III/IV disease, undergoing combined chemo-radiotherapy with cisplatin were treated with escalating doses of *i.t.* T-VEC [19]. These patients were previously untreated. At surgery, 93% of patients had a pathological complete response (CR). Once again, despite HSV-seropositivity, HSV was found in both injected and non-injected areas of tumour.

### 6.5. Oncolytic Virus and Anti-Angiogenic Therapy

Sunitinib and sorafenib (small molecule inhibitors of a number of anti-angiogenic proteins) and bevacizumab (an anti-vascular endothelial growth factor (VEGF) monoclonal antibody), have also been investigated in combination with many OVs with some encouraging results. In a murine breast cancer model of *i.t.* HSV in combination with bevacizumab, there was evidence of viral distribution to breast cancer cells resulting in decreased tumour size [91]. Clinically, bevacizumab has shown some promise in HER-2 negative and triple-negative breast cancer (TNBC), which is hormone receptor-negative and, therefore, resistant to these therapies [92,93,94].

GLV-1h164 is a modified VV encoding anti-VEGF antibody. In TNBC, GLV-1h164 decreased mean tumour volumes and increased tumour cytotoxicity in a murine model; decreased angiogenesis and decreased vascular flow to tumours was also apparent [95].

Sunitinib and VSV treatment of prostate and breast cancer cell lines resulted in increased cell death [96]. *In vivo,* increased viral replication was seen and the effect was similar in both immuno-competent and nude (immuno-deficient) mice. The explanation for this may be twofold: by a synergistic anti-angiogenic effect (VSV also can destroy tumour vasculature [97]) and by inhibition of PKR by sunitinib, which can confer resistance to viral replication (and therefore decrease efficacy of oncolytic viral therapy). Interestingly, in a separate *in vivo* model, conditioning with VEGF prior to treatment with reovirus led to enhanced anti-tumour effects and viral recovery from target tissues [98].

Although there is pre-clinical evidence for the use of OVs and anti-angiogenic therapy, questions have been raised about whether decreased neo-angiogenesis will limit delivery of virus to tumours in human participants. OVs themselves can have anti-angiogenic properties [99]. A number of early phase clinical trials investigating this synergistic effect are open at the moment and the results are awaited with interest.

### 6.6. Oncolytic Virus and Monoclonal Antibodies

Monoclonal antibodies, such as trastuzumab, act via multiple mechanisms: firstly, by a direct effect on binding to a target ligand, disrupting intracellular signalling pathways (in this case HER-2) and secondly, via antibody-dependent cellular cytotoxicity (ADCC). Indeed, activation of NK cells with reovirus has been shown to support ADCC in CLL cell lines treated with the anti-CD20 antibody rituximab [46]. Trastuzumab has also shown potential when combined with reovirus. In *in vitro* and *in vivo* models, combination therapy increased the cytotoxic and anti-tumour effects mediated, to some extent, by TRAIL [62,100].

### 6.7. Oncolytic Virus and Cytokines

It is recognised that OVs exert an immunogenic, in addition to a directly cytotoxic, effect. Combination therapy with stimulatory cytokines aims to exploit and build on this to augment efficacy by enhancing response against TAA. Adenovirus expressing IL-12, for example, has been shown to have greater therapeutic potential *in vivo* and lead to increased levels of IL-12 [101]. This targeted approach also has the potential to limit toxicity that has been seen with systemic cytokine therapy.

A similar effect has been seen in an ovarian cancer murine model treated with HSV expressing murine GM-CSF, the hypothesis being that the GM-CSF will act as an immune stimulus and potentiate the oncolytic effect of HSV [102]. This was supported by increased infiltration of CD4+ and CD8+ T cells into the tumour microenvironment, in association with decreased tumour growth and increased survival compared to parental HSV. It is noteworthy that the current lead clinical OV agent, T-VEC, encodes GM-CSF.

Trials using JX-594 (VV encoding GM-CSF) are ongoing. It has shown some promise in patients with hepatocellular carcinoma (HCC) and a pivotal phase III trial of sorafenib ± JX-594 in this patient group is ongoing [20,66]. A translational study evaluating the immune response to JX-594, including in HCC, has also just opened for recruitment.

### 6.8. Oncolytic Virus and Chemokines

One of the barriers to overcome in OV therapy is the immunosuppressive nature of the tumour microenvironment. By arming OVs with chemokines, which can attract immune cell populations to the tumour, there is potential for this effect to be dampened. In a murine model of CRC, VV expressing RANTES has been shown to increase the expression of Th1 cytokines (IFN-γ, IL-2, CXCL-10) and infiltration of CD4+ T cells, DCs, and NK cells into this environment [103]. This corresponded with an increased anti-tumour immune response and therapeutic effect.

Similarly, VV expressing CXCL-11, another chemoattractant, was able to increase IFN-γ producing CD8+ T cells in a mouse mesothelioma model and led to a decrease in immunosuppressive modulators such as transforming growth factor (TGF)-β [104]. Interestingly, in both cases there was no evidence of an increase in MDSC [103] or Treg populations [103,104]. Adenovirus armed with RANTES and IL-15 has also been used *in vivo* to enhance the infiltration of tumour-directed chimeric antigen receptor (CAR)-T cells, used in adoptive T cell therapy [105]. Increased levels of IL-15 and RANTES were found at the tumour site in mice inoculated with neuroblastoma cells. This translated to increased T cell infiltration and increased survival. Whether these findings translate to a clinical setting has not been thoroughly investigated.

### 6.9. Oncolytic Virus and Oncolytic Virus

Although not yet used clinically, the idea of OV/OV combination is an attractive one, with potentially limited toxicity and the ability to minimise problematic NAbs if viruses are alternated during therapy. A synergistic effect may be achieved if the viruses in question have different modes of action. In a number of pre-clinical tumour models, Le Boeuf *et al.* [106], showed some evidence of synergy when combining VV with VSV. Viral replication and infection *in vitro* were both increased, with some suggestion of activity in clinical tumour samples *ex vivo*.

So far, combination virotherapy has not translated into meaningful clinical signals, although there is a paucity of evidence. In 22 patients treated with two OVs with different capsid proteins, including adenovirus encoding GM-CSF, there was found to be no survival advantage over three sequential doses of adenovirus [3].

## 7. Efficacy of Oncolytic Viruses

There is extensive literature regarding the pre-clinical activity of a multitude of OVs but evidence of clinical efficacy is still emerging. While there are case reports of significant responses to OVs in patients with disseminated disease [107], only a small number of phase II or III trials have been published.

The most recent of these and, as yet, the first trial of oncolytic therapy to show tangible benefit, is the phase III trial of T-VEC. In this trial of 436 patients randomised to receive either virus or single agent GM-CSF, T-VEC showed evidence of clinical efficacy in patients with metastatic malignant melanoma [2]. Overall response rate (ORR) for the T-VEC arm was 26.4% *vs.* 5.7% in the GM-CSF alone arm, with durable response rates (DRR, objective response lasting over 6 months) of 16.3% *vs.* 2.1%, respectively. OS in the group receiving T-VEC was 23.3 months *vs.* 18.9 months (*p* = 0.051). A more noticeable difference was evident in patients who were treatment naïve, suggesting there may be an altered immune response in patients who have received previous lines of chemotherapy.

In the phase II trial of reovirus given *i.v.* to 21 patients with metastatic melanoma, time to progression was 45 days, with a median OS of 165 days [16]. Reovirus was generally well tolerated. The main toxicities, as had been previously reported, were fatigue, nausea, myalgia, and fever. Over 95% of the patients had received previous lines of systemic therapy and over half had visceral disease. There were no objective responses noted in terms of CR and PR but it should be noted that B-rapidly accelerated fibrosarcoma (*BRAF*) mutations were less common than generally expected, in only 2/13 evaluable patients. All patients had NAbs to reovirus at baseline. Interestingly, it was the two patients who had amongst the highest NAb titres in whom replicating virus was found after tumour resection.

Intratumoural ONYX-015 adenovirus can selectively replicate in *p53*-deficient cancer cell lines; combination with cisplatin and 5-FU for recurrent squamous cell carcinoma of the head and neck showed some promise in a phase II trial [108]. In an “intent to treat” analysis of 37 patients, in those whom a response could be evaluated, just over half had an objective response. Again, no link between disease response and viral antibody levels at baseline was reported.

JX-594 has been primarily used in patients with HCC; in a phase II dose-finding study, “low” (10^8^ plaque forming units, pfu) or “high” (10^9^ pfu) dose JX-594 was given *i.t.* to 30 patients with advanced HCC. Median survival was significantly higher in the “high” dose group: 14.1 months *vs.* 6.7 months (*p* = 0.02) [20]. As before, this did not correlate with baseline NAb titre. Unfortunately, the phase IIb trial of JX-594 *vs.* best supportive care (BSC) in patients with HCC, who have failed sorafenib therapy, did not reach its primary end-point of increased OS [109]. JX-594 has also been used sequentially with sorafenib in three patients with HCC as an extension to *in vivo* work by the same group [110]. These patients were treated with sorafenib after progressing following treatment with JX-594: all three were observed to have increased tumour necrosis on imaging after starting sorafenib, although this has also been reported with sorafenib monotherapy [111]. As mentioned previously, a phase III trial of sorafenib with or without JX-594 in patients with HCC is in development [66].

PROSTVAC-VF, an engineered poxvirus (consisting of sequential treatment with vaccinia and fowlpox viral vectors encoding transgenes for PSA and other co-stimulatory molecules), has entered phase III trials in men with metastatic castrate-resistant prostate cancer (NCT01322490). The phase II data of PROSTVAC-VF with GM-CSF given to 125 patients, showed an 8.5 month OS benefit when compared to controls (25.1 months *vs.* 16.6 months, respectively) [22].

In melanoma, coxsackie virus A21 (CAVATAK^®^) has shown some promise. The CAVATAK^®^ in Late stage Melanoma (CALM) study reached its primary endpoint with 22/57 evaluable patients maintaining a CR, PR or SD by Response Evaluation Criteria In Solid Tumours (RECIST) six months after initiation of *i.t.* treatment. The one-year survival rate was 73.3% [7].

## 8. Timing of Therapy

It is a truth universally acknowledged that early phase trials of anti-cancer therapy involve patients who have already had a long clinical course and multiple lines of treatment. If a novel agent yields promise in this group, then dogma dictates that we work backwards to earlier lines of palliative treatment until we eventually reach the adjuvant and the “potentially cured” setting. For this reason, data surrounding the use of OVs earlier in the treatment pathway is, as yet, sparse. There is a small amount of data regarding the use of melanoma antigen specific vaccines in the adjuvant setting [85].

NK cells, with the potential to be cytotoxic to errant tumour cells which can escape during surgery, have been shown to have diminished function post-operatively. This leads to a window of opportunity, whereby the administration of an OV, such as vaccinia, can be used to override this suppression and prevent metastatic disease. This was observed *in vivo* in mice and potentially in patients receiving the modified vaccinia virus, JX-594 [112]. Influenza vaccine given peri-operatively has also been shown to reverse the post-operative suppression of NK cell-mediated cytotoxicity and activity, with evidence of increased expression of the activation marker CD69, in cells from both healthy donors and cancer patients [113]. In a mouse melanoma model this translated to a decreased number of metastases following surgery. Similar findings have been seen using the maraba virus [114].

The concern, with any neo-adjuvant or peri-operative intervention, is that associated side-effects may render the patient too unwell for curative therapy or infer increased complications. As agents that have low levels of toxicity, this potentially opens another avenue for the use of OV in the clinic. An important point to bear in mind, however, is that the immune response to OV may well be different in the absence of active, measurable disease, as is the case in the adjuvant treatment setting.

## 9. Choosing your Patient: The Search for Biomarkers

After discussing the “what”, “why”, “how”, and “when”, consideration really must be given to the “who” of oncolytic virotherapy. Although numerous avenues have been investigated in terms of both predictive and prognostic biomarkers, particularly those which form part of the immune response, generic consideration is needed in patient selection in these groups: patients entering trials with OVs are likely to have advanced disease and may have been heavily pre-treated. This may, in turn, bring into question whether they have a “normally” functioning immune system and to what extent results may be extrapolated to the rest of the patient population.

One of the challenges in the use of OV, either as monotherapy or in combination with other agents, is the identification of predictive biomarkers to identify patients who are likely to respond. Immune biomarkers already postulated, in addition to NAb levels, include TILs, Treg activation, PD-1 or PD-L1 expression, as well as other numerous markers of the innate or adaptive immune response to viral therapy, including epitope spreading [115].

The immunoglobulin-like transcript (ILT)-2 gene has been shown to be down-regulated in response to VV [116]. ILT-2 is expressed on the surface of T cells and has been shown to suppress T cell function and activation. It is also expressed at higher levels on T cell subsets in patients with metastatic melanoma, including CD4+ and CD8+ cells and Tregs [116]. There is decreased expression of ILT-2 on the T cells of patients with melanoma who respond to vaccinia therapy, which may indicate its potential use as a predicative marker of response.

Cathepsins B and L are proteases involved in the infection of cells by oncolytic reovirus. They have also been investigated and found to be present in higher levels in cells lines which are more vulnerable to reovirus-mediated cell death [117]. Fc-gamma (Fcγ) receptor polymorphisms have also been explored and, in particular, whether they confer any prognostic benefit in patients treated with adenovirus [118].

Neutrophil-lymphocyte ratio (NLR) and how it changes in response to treatment, has been shown to be prognostic in a number of tumour sites as well as in unselected groups of patients entering early phase clinical trials [119,120,121,122]. This has not been studied specifically in patients treated with OV. Low total lymphocyte count (TLC) has also been shown to have some prognostic significance in patients treated with GM-CSF-secreting pancreatic tumour vaccine (GVAX), where TLC was linked to both overall and disease-free survival [123].

## 10. Oncolytic Virus Therapy: The Health and the Harm

Everyone working in viral therapy should know the name Jesse Gelsinger. In 1999, the 18-year-old received a genetically-modified adenovirus via hepatic artery infusion as part of a clinical trial for a non-malignant genetic disease. Less than 24 h after dosing, Mr. Gelsinger developed a systemic inflammatory response syndrome and disseminated intravascular coagulation (DIC), followed by multi-organ failure [124]. He died four days later. While the effect on his family was devastating, there was also a profound effect on the medical team caring for him [125]. Similar, less severe, toxicity was seen in another trial of adenovirus, also given by hepatic artery infusion. On this occasion, the vector in question was Onyx-015 in combination with 5-FU in patients with CRC liver metastases [60]. In this phase II trial, one patient developed a systemic inflammatory response, with evidence of liver impairment and raised levels of the pro-inflammatory cytokine IL-6, as seen in Mr. Gelsinger’s case. There was also a possible treatment-related death in the phase I trial of VSV-hIFN-β (VSV with an IFN-β gene insert), where a patient with CRC developed hepatorenal syndrome and died 13 days after treatment [23]. These examples illustrate some of the difficulties in the development of immunotherapeutics, in particular the inter-patient variability and unpredictability of the immune response. It is important to keep in mind that all clinical interventions have consequences: we try to make calculated and intelligent decisions but there remains an element of risk, particularly in early phase clinical trials of novel therapy. Although we should remain cautious, overall in OV therapy there have been relatively few severe side effects. In the phase I study of gemcitabine and reovirus, there were three DLTs: two patients with asymptomatic rise in hepatic transaminases (which spontaneously resolved) and one patient with a troponin rise but no cardiac sequelae [13].

There has been some work looking into moderating the inflammatory response to OV. It is a fine line to tread between controlling potentially dangerous toxicity and damping down the anti-tumour immune effect: a line that has had to be carefully monitored in the development of checkpoint inhibitors. β3 integrins on the cell surface are thought to play a role in inducing cytokine-mediated inflammation after adenoviral therapy [126]. Inflammatory cytokine production can be decreased by co-administration of a β3 integrin inhibitor in a murine model of ovarian cancer, apparently without inhibiting viral-mediated oncolysis [126]. What effect this has on the adaptive anti-tumour response is unclear. In an era where interest in immune checkpoint inhibitors seems to be increasing exponentially, serious consideration should be given to the consequences of giving an OV in combination with these agents. In reality, fears of patients having an overwhelming and uncontrolled immune response to OV in this context seem to be unfounded: there were no DLTs reported in a primary analysis of 18 patients receiving combination therapy with T-VEC and anti-CTLA-4 antibody [17]. Early phase trials of T-VEC in combination with ipilimumab and pembrolizumab are currently open to recruitment (NCT01740297 and NCT02263508, respectively) with larger trials, as mentioned above, in development.

## 11. Conclusions

The inherent difficulty in the transition of therapeutic agents with pre-clinical in vivo potential into a clinical setting is as wide and varied as the difference between the mouse and the patient. Nowhere is this truer than in the context of immune therapy, particularly virotherapy. The most legitimate definition of “personalised medicine” in this context must, therefore, be this: the right virus, for the right patient, at the right time. As it becomes more evident that OVs trigger an anti-tumour immune response as well as a direct cytotoxic effect, we must endeavour to better understand these beneficial mechanisms in a clinical (rather than murine) setting. We may otherwise run the risk of wasting this potential and relegating OV therapy into the category of “what might have been”. Nevertheless, with increasing momentum across the entire field of cancer immunotherapy and encouraging emerging data supporting the safety and potential efficacy of OVs, there is a significant chance that viruses may become an entirely new modality for the treatment of malignancy before too long.
